# Syndrome de Bean chez l'enfant: à propos de deux cas

**DOI:** 10.11604/pamj.2017.28.102.11109

**Published:** 2017-10-04

**Authors:** Achraf El Bakkaly, Fouad Ettayebi, Houda Oubeja, Mounir Erraji, Hicham Zerhouni

**Affiliations:** 1 Service des Urgences Chirurgicales Pédiatriques, CHU Ibn Sina, Faculté de Médecine Mohammed V, Rabat, Maroc

**Keywords:** Angiomatose, syndrôme de Bean, chirurgie, Angiomatosis, Bean's syndrome, surgery

## Abstract

L'angiomatose diffuse ou syndrome de Bean est une entité rare caractérisée par des malformations veineuses essentiellement cutanées et digestives pouvant se compliquer d'hémorragie de gravité variable. Notre travail intéresse l'étude de deux enfants, l'un âgé de 5 ans et l'autre de 9 ans et demi atteints d'angiomatose diffuse suivis au service des urgences chirurgicales pédiatriques depuis des années. Le diagnostic a été évoqué devant des rectorragies et/ou des mélénas occasionnant une anémie sévère nécessitant des transfusions régulières chez nos deux patients ainsi que l'apparition des angiomes cutanés au niveau des membres. Les explorations radiologiques ont révélé la présence au niveau du jéjunum et l'iléon des multiples lésions correspondant à une angiomatose grêlique diffuse pour l'enfant de 9 ans et demi; elles n'ont pas retrouvé de localisations abdominales pour l'enfant de 5ans. Les deux malades ont été admis au bloc opératoire avec individualisation à l'exploration des angiomes dont certains saignaient activement. Une résection par entérotomie a été réalisée. Les suites ont été marquées par l'arrêt des saignements. L'intérêt de notre travail est de mettre le point sur cette entité pathologique rare, ainsi que le bénéfice du traitement chirurgical pour le contrôle des complications de cette pathologie et pour la diminution de la fréquence des transfusions.

## Abstract (en)

Diffuse angiomatosis or Bean's syndrome is a rare disease characterized by venous malformations mainly involving the skin and the digestive tract which can result in hemorrhage of variable severity. This study reports the case of two children aged 5 and 9 and a half years respectively with diffuse angiomatosis who had been treated in the Department of Emergency Paediatric Surgery over the years. The diagnosis was based on rectal bleeding and/or melenas causing severe anemia requiring regular transfusions in both patients as well as skin angiomas occurrence at the level of the limbs. Radiological evaluation showed the presence of multiple lesions at the level of the jejunum and ileum consistent with small intestinal angiomatosis in the child aged 9 and a half years. It didn't show abdominal lesions in the child aged 5 years. The two patients were admitted to the operating block. Angiomas were surgically individualized. Some of them were actively bleeding. Enterotomy was performed. Postoperative sequelae was marked by the stop of the bleedings. This study aims to update the current understanding of this rare pathology as well as the benefit of surgical treatment in controlling the complications caused by this pathology and in reducing the frequency of transfusions.

## Introduction

Le syndrome de Bean est une maladie rare caractérisée par des malformations veineuses essentiellement cutanées et digestives, dont les complications hémorragiques peuvent engager le pronostic vital [1]. Ces malformations veineuses sont multifocales et touchent le plus souvent la peau et le tractus digestif, mais peuvent également être localisées au niveau du cerveau, des reins, des poumons, des yeux, des os et même d'autres organes. Les lésions sont généralement présentes dès l'enfance mais peuvent également se développer plus tard dans la vie [2]. De diagnostic difficile, nous rapportons à travers ce travail l'observation de deux enfants suivis pour syndrome de Bean, entité vasculaire rare à ne pas méconnaître, dont le traitement chirurgical a permis de contrôler les lésions intestinales responsables d'hémorragies et d'améliorer ainsi la qualité de vie de nos patients.

## Patient et observation

### Observation n°1

Il s'agit de S.M. enfant âgé de 5 ans, deuxième d'une fratrie de deux, bien vacciné selon le programme national de vaccination, ayant comme antécédents une communication interventriculaire trabéculaire, traitée par cerclage de l'artère pulmonaire à l'âge de 7 mois. Nous signalons une notion d'hospitalisation à trois reprises au service pour prise en charge de syndrome anémique non étiqueté jusque-là. Le début de la symptomatologie remonte à l'âge de 3ans et 8 mois, par un saignement après son cerclage de l'artère pulmonaire, ayant nécessité une première transfusion. Par la suite, l'enfant a développé un syndrome anémique pour lequel il a été mis sous traitement martial, en plusieurs cures sans résultats satisfaisants, d'où son orientation vers l'hôpital d'enfants de Rabat pour bilan étiologique. Lors de sa première hospitalisation, l'enfant se présentait pour un syndrome anémique franc, avec pâleur cutanéomuqueuse et asthénie avec des chiffres de numération objectivant une anémie hypochrome microcytaire hyposidéremique, pour lesquels il a bénéficié d'une transfusion de culots globulaires. Son examen clinique trouvait un enfant pâle asthénique avec à l'examen cardiaque un souffle en jet de vapeur.

**Figure 1:**
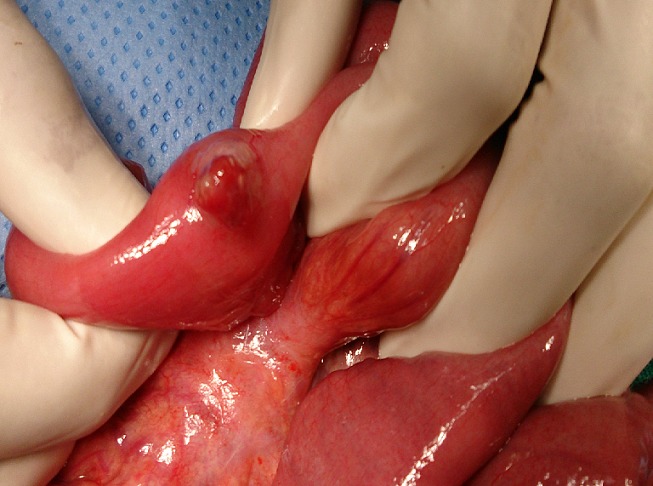
Image en per opératoire montrant un angiome saignant activement au niveau de l'iléon

Lors de sa deuxième hospitalisation pour le même tableau clinique, pour lequel il a bénéficié d'une nouvelle transfusion, le bilan étiologique réalisé dans le cadre de la recherche d'un foyer de saignement occulte s'est avéré jusque-là non concluant. Par ailleurs, l'examen clinique de l'enfant a objectivé une douleur au niveau du coude et de la plante du pied droits. L'exploration échographique couplée au doppler des deux foyers a objective la présence d'angiomes au niveau des deux sièges sans autres anomalies.Citons que par ailleurs, l'examen clinique ne rapporte pas la présence d'angiomes cutanés. Un bilan d'extension à la recherche d'autres localisations, soit une imagerie par résonnance magnétique cérébrale, a objectivé la présence d'une malformation vasculaire temporale droite. L'échographie abdominale ne retrouve pas de localisations intra abdominales. Le reste des explorations étiologiques parallèles revenaient tjrs normales. L'enfant revient 20 jours après dans un tableau d'anémie sévère avec méléna nécessitant une transfusion en urgence, compliquée par l'apparition de rectorragies d'importante abondance le jour même, avec à l'examen un abdomen souple non douloureux. Un doppler abdominal réalisé en urgence parle d'une invagination grêlo-grêlique, secondaire a une masse, probablement un angiome vu le contexte clinique. Uneéchographie cardiaque réalisée aussi vu le contexte particulier de l'enfant a objectivé un état stable permettant la prise en charge chirurgicale. L'enfant est transféré par la suite au service des urgences chirurgicales pour une exploration laparoscopique convertie en laparotomie après la découverte de plusieurs angiomes grêliques (Figure 1). Une résection des angiomes a été réalisée. Nous notons par ailleurs l'absence d'invagination, de diverticule du Meckel ou de masse intra abdominale. Les suites post opératoires étaient sans incidents, avec la reprise d'un bon état général et la disparition des rectorragies. L'étude anatomopathologique de la pièce opératoire parle d'un hémangiome grêlique. Avec un recul de 3 ans, l'enfant n'a plus présenté d'épisodes de saignement. Toujours sous traitement martial, il garde toujours des chiffres d'hémoglobine de contrôle corrects.

### Observation n°2

Il s'agit de l'enfant O.J âgé de 9 ans et demi, l'aîné d'une fratrie de deux, bien vacciné selon le programme national d'immunisation (PNI), ayant comme antécédents une notion d'hospitalisations à 4 reprises au service pour un syndrome anémique non étiqueté jusque-là. Le début de la symptomatologie remonte à l'âge de 4 ans où l'enfant a présenté des rectorragies nécessitant une transfusion, il a été mis sous traitement martial sans réel rétablissement. Lors de ses hospitalisations, l'enfant a présenté un syndrôme anémiquefranc, avec un tableau clinique fait d'une pâleur cutanéo-muqueuse, asthénie et un souffle systolique à l'auscultation cardiaque. Une numération formule sanguine (NFS) a été réalisée objectivant une anémie profonde hypochrome microcytaire avec des réserves ferriprives effondrées, l'enfant a bénéficié d'une réanimationhydro-électrolytique et des transfusions de culots globulaires. Par ailleurs, à l'âge de 8 ans, l'enfant a présenté des douleurs au niveau du coude et de la plante du pied droits.

**Figure 2:**
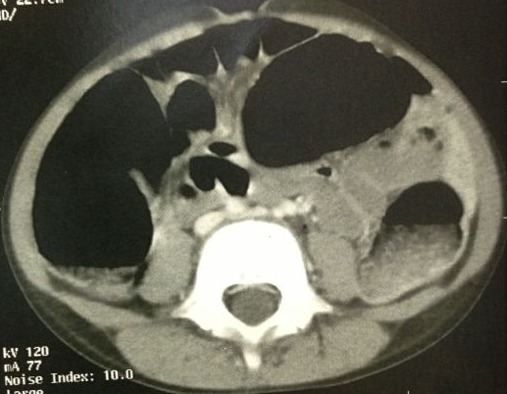
TDM abdominale de notre patient montrant la présence au niveau du jéjunum d'une masse d'environ 3 cm hyper-vasculaire compatible avec un hémangiome

Dans le cadre d'un bilan d'extension, une tomodensitométrie et une imagerie par résonance magnétique cérébrale sont réalisées, objectivant la présence d'une malformation vasculaire cérébrale temporale droite. L'échographie abdominale n'a pas trouvé de localisations intra-abdominales. Le reste des explorations étiologiques parallèles sont revenues normales notamment une scintigraphie abdominale et une fibroscopie oeso-gastro-duodénale. L'enfant est revenu un mois après, dans un tableau d'anémie sévère avecdes rectorragies importantes nécessitant une réanimation avec une transfusion enurgence. L'examen clinique à l'admission a retrouvé un enfant asthénique avec pâleur cutanéomuqueuse et une tachycardie à 130 battements par minute. La tension artérielle était normale. L'écho-doppler abdominale a révélé la présence d'un boudin d'invagination grêlo-grêlique, secondaire à une masse, probablement un angiome vu le contexte clinique. La tomodensitométrie abdominale est demandée, montrant la présence au niveau du jéjunum d'une masse d'environ 3cm hyper-vasculaire compatible avec un hémangiome et présence de deux autres lésions pouvant correspondre à une angiomatose grêlique diffuse (Figure 2).

**Figure 3:**
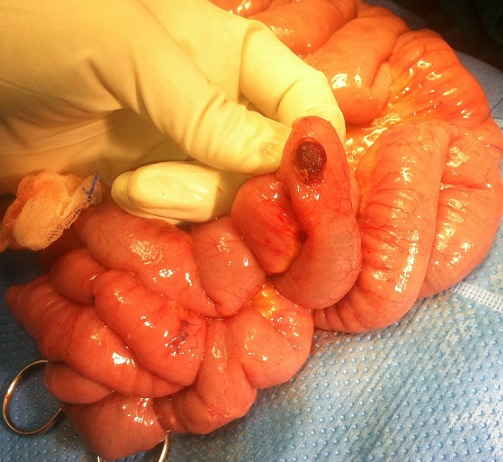
Image en per opératoire du gros angiome au niveau grêlique saignant (entérotomie)

Après la réalisation d'un bilan pré-anesthésique notamment une échographie cardiaque éliminant une contre-indication à la chirurgie, l'enfant est transféré au service des urgences chirurgicales pédiatriques pour une exploration chirurgicale. L'exploration chirurgicale par voie coelioscopique est faite, l'examen du grêle a objectivé une angiomatose intestinale diffuse. Après extériorisation du grêle, le geste chirurgical a consisté en une électrocoagulation des petites lésions et une résection limitée du gros angiome suivie de sutures transversales (Figure 3). L'intervention a duré 1h35min. Notre malade a bénéficié d'une exploration chirurgicale par voie coelioscopique pour une évaluation initiale aussi bien diagnostic que thérapeutique, cette voie d'abord offre l'élégance, l'efficacité, l'économie et le confort. L'étude anatomopathologique de la pièce opératoire parle d'une malformation veineuse de localisation grêlique. Les suites opératoires sont simples, le malade a quitté le service dans les 72 heures qui suivent. Le suivi à long terme a objectivé une récidive des rectorragies qui a nécessité un autre séjour hospitalier dans notre service, une deuxième exploration laparoscopique est faite avec une cure chirurgicale des deux autres gros angiomes. Les suites ont été marquées par l'arrêt des saignements. L'enfant est suivi depuis lors régulièrement en consultation, avec un recul de 3 ans, ne présentant plus d'épisodes de saignement et il a été mis sous traitement martial. Les chiffres de l'hémoglobine de contrôle sont corrects. L'association de l'atteinte cutanée et digestive de ces malformations veineuses fait évoquer en premier le syndrôme de Bean, qui est retenue comme diagnostic définitif pour nos deux malades.

## Discussion

Le syndrôme « bluerubber-blebnaevus » ou angiomatose diffuse a été signalé pour la première fois en 1860, chez un patient qui a présenté une asphyxie suite à une hémorragie aigue d'une tumeur parotidienne. Il avait aussi des angiomes multiples au niveau cutané etdigestif. Appelé aussi syndrôme de Bean, c'est cet auteur qui en 1958, décrit les lésions cutanées compressibles à la palpation en « Tétine de caoutchouc » [3]. Plus de 200 cas ont été décrits, mais la prévalence reste inconnue. Ce syndrôme de Bean est une malformation veineuse disséminée et sporadique qui pourrait être familiale avec une transmission autosomique dominante secondaire à une mutation sur le chromosome 9p [1], mais il semble qu'il y ait eu confusion dans la littérature avec les malformations veineuses cutanéo-muqueuses sans localisation viscérale et dont la forme familiale est liée au gène muté « TIE2 »; le syndrome de Beann'est pas lié au gène muté « TIE2 ». Les signes cliniques principaux découlent du saignement aigu ou chronique des multiples lésions du tractus digestif qui siègent surtout au niveau de l'intestin grêle. Les saignements aigus se présentent comme des hématémèses, des mélénas ou des rectorragies. Les lésions sont cutanées et viscérales (atteinte digestive gastro-intestinale responsable d'hémorragies [4] et atteintes urinaire, hépatique, splénique, cérébrale, pulmonaire, etc.). Les atteintes extra-digestives peuvent être responsables d'une déformation orthopédique [5], une démence [6], une embolie pulmonaire, une hypertension de l'artère pulmonaire, un hémothorax, un hémopéricarde [7], une exophtalmie [8], une thrombopénie, une coagulopathie de consommation chronique. Les lésions cutanées peuvent être maculaires, papuleuses, nodulaires oupédonculés, rougeâtres ou violacées. Elles sont multiples et le plus souvent nonsaignantes [9, 10]. On peut les classer en 3 types [11]: 1) Le type 1: il s'agit d'une grande lésion déformante qui peut obstruer l'organe adjacent. 2) Le type 2: c'est le type le plus fréquent, il est de couleur bleue froissée en« tétine de caoutchouc » recouvert de peau [12]. 3) Le type 3: c'est une lésion maculaire ou papulaire de couleur bleu-noirequi peut blanchir à la pression. Ces lésions cutanées peuvent être présentes à la naissance ou apparaître au fil des années [9-11].

Les angiomes cutanés peuvent devenir douloureux, cette douleur est le fait de la mise en tension de ces lésions dans certaines positions, mais aussi de thromboses localisées, à l'origine des phlébolithes qui sont des calcifications rondes pathognomoniques des malformations veineuses et bien visibles sur les radiographies [9, 10]. L'hémorragie digestive est la complication la plus fréquente de l'atteinte intestinale dans le syndrome de Bean, mais une invagination intestinale est aussi décrite dans quelques publications [13] comme c'est le cas pour nos deux patients. Les méthodes diagnostiques comprennent la numération sanguine, l'endoscopie, l'échographie, le scanner, l'imagerie par résonance magnétique et l'histologie. Le syndrome de Bean est une forme clinique rare de malformations veineuses dont le traitement est à la fois médical et chirurgical. Le pronostic de syndrome de Bean dépend des complications essentiellement hémorragiques et de la localisation de ces angiomes par rapport aux organes vitaux. En l'absence d'un traitement approprié, les patients atteints souffrent d'une anémie due aux saignements chroniques qui nécessite l'administration de fer et de transfusions à répétition. La transformation maligne des lésions n'a, jusqu'à présent, pas été décrite. En l'absence de saignement intestinal important, un traitement minimum est suffisant (supplémentation en fer et/ou transfusions). L'administration au long cours d'octréotide réduit les pertes sanguines du syndrôme de Bean et d'autres lésions vasculaires gastro-intestinales. Dans notre expérience, la chirurgie avec exérèse des angiomes intestinaux par voie laparoscopique ou classique, semble être le moyen efficace pour améliorer le pronostic des enfants atteints de ce syndrôme et réduire ainsi la fréquence des transfusions, c'est le cas pour notre deuxième patient dont on a pu contrôler l'hémorragie causée par les angiomes intestinaux, grâce à deux interventions chirurgicales, lui évitant depuis lors des transfusions répétitives. Nos résultats corroborent parfaitement avec celles de Zhi Li [14] qui admet que l'éradication chirurgicale des angiomes intestinaux est le moyen efficace pour traiter l'angiomatose de Bean. Ainsi, sur une étude faite sur 2 malades, l'auteur démontre que la chirurgie apporte le bénéfice de contrôler des saignements graves chroniques sur des angiomes intestinaux quel que soit leur nombre ou leur emplacement et ceci par résection de toutes les lésions responsables de saignement ainsi que des segments digestifs sièges de lésions de viabilité douteuse. Ce traitement couplé à une supplémentation en fer seulement, a permis d'améliorer les chiffres d'hémoglobine chez les 2 patients sans être obligé à leur refaire des transfusions. Seulement un patient a été réadmis au bloc avec exérèse des angiomes au niveau du duodénum et du côlon avec une nette amélioration clinique et des chiffres d'hémoglobine post-opératoires.

Aucun consensus n'a été établi sur le traitement de cette angiomatose diffuse. Puisque le saignement chronique des angiomes au niveau gastro-intestinal a persisté durant toute la vie des patients atteints de cette entité pathologique. Un grand choix de stratégies thérapeutiques pharmacologiques a été proposé, notamment l'interféron antiangiogenic. Cependant, ces agents n'ont pas été démontrés pour être fiables et efficaces pour arrêter le saignement et contrôler ainsi ces angiomes gastro-intestinaux [15, 16]. Récemment, Fishman et d'autres chercheurs ont conclu que les lésions de syndrôme de Bean sont des malformations vasculaires congénitales plutôt que des tumeurs de prolifération. En se basant sur cette théorie, Fishman a soigné 10 patients de syndrôme de Beanpar la chirurgie, une moyenne de 137 lésions ont été réséqués pour l'ensemble des patients, avec un peu de répétition des saignements sans anémie grave chez un seul patient, et d'avantage d'intervention chirurgicale chez deux patients [17]. Ces deux cas repris chirurgicalement ont également démontré que l'éradication chirurgicale pour les lésions gastro-intestinales de cette angiomatose était importante et efficace pour arrêter définitivement le saignement de ces angiomes gastro-intestinaux. Dans notre expérience, nous croyons que le saignement chronique grave des angiomes digestifs de l'angiomatose diffuse peut être jugulé avec succès par la résection de ces angiomes, indépendamment de leur nombre ou de leur emplacement. Il est peu susceptible de les traiter par les autres thérapeutiques pharmacologiques. Cette approche chirurgicale effective peut aider à éviter les ravages de l'anémie chronique, y compris la fatigue, la surcharge de fer, et les risques potentiels des transfusions sanguines répétitives comprenant le risque de contamination par hépatite et accidents allergiques. L'intervention chirurgicale est la plus bien projetée quand une indication de transfusion devient évidente. Une fois une approche effective est choisie, une visualisation complète du tractus gastro-intestinal entier de la bouche à l'anus doit être exécutée. L'éradication complète peut éviter le besoin de reprises chirurgicales. La résection inachevée de n'importe quelles lésions données peut tenir compte du risque de récidive et du saignement récurrent. Bien qu'extrêmement pénibles, les principes standards et les techniques chirurgicaux combinés avec la gestion anesthésique soigneuse permettent un très bon résultat avec une morbidité minimale. Les patients avec un grand nombre de lésions peuvent avoir besoin des procédés chirurgicaux étagés. Néanmoins, cette approche améliore spectaculairement la santé et le bien-être des patients présentant le syndrôme de Bean. Toutefois, l'entéroscopie per-opératoire est actuellement la procédure diagnostique et/ou thérapeutique définitive puisqu'elle permet le traitement immédiat des lésions par coagulation endoscopique ou par excision chirurgicale complète. L'indication pour cette intervention peut être posée à partir des résultats de l'endoscopie par capsule qui permet également de contrôler ensuite les résultats du traitement [18]. Cette procédure bien qu'extrêmement bénéfique, est

## Conclusion

L'angiomatose diffuse est une malformation vasculaire rare [19]. Elle est difficile à diagnostiquer en raison de sa faible fréquence. Le caractère disséminé des lésions rend un traitement radical souvent difficile au cours du syndrome de Bean [2]. Le pronostic après exérèse des angiomes intestinaux est très bon avec réduction de fréquence des transfusions. Cependant, le traitement endoscopique s'est montré comme une modalité thérapeutique efficace à côté de la chirurgie.

## Conflits d'intérêts

Les auteurs ne déclarent aucun conflit d'intérêts.
